# Supplementary material for: Phables: from fragmented assemblies to high-quality bacteriophage genomes
Source: Bioinformatics. 2023 Sep 21;39(10):btad586. doi: 10.1093/bioinformatics/btad586 (PMC10563150; doi:10.1093/bioinformatics/btad586)
Supplement: btad586_Supplementary_Data [file btad586_supplementary_data.zip › Phables_Supplementary_Material.pdf]

Phables: from fragmented assemblies to high-quality bacteriophage genomes

Supplementary Material

Vijini Mallawaarachchi[1], Michael J. Roach[1], Przemyslaw Decewicz[2,1], Bhavya Papudeshi[1], Sarah K. Giles[1], Susanna R. Grigson[1], George Bouras[3], Ryan D. Hesse[1], Laura K. Inglis[1], Abbey L. K. Hutton[1], Elizabeth A. Dinsdale[1] and Robert A. Edwards[1]

[1]Flinders Accelerator for Microbiome Exploration, College of Science and Engineering, Flinders University, Bedford Park, 5042, South Australia, Australia

[2]Department of Environmental Microbiology and Biotechnology, Institute of Microbiology, Faculty of Biology, University of Warsaw, Warsaw 02-096, Poland

[3]Adelaide Medical School, The University of Adelaide, North Tce, Adelaide, 5000, South Australia, Australia

Keywords: bacteriophages, metagenomics, genomics, assembly graphs

1. Comparison of existing tools to identify phage sequences

Table S1. Feature comparison of available tools to identify/bin sequences from phage origin in metagenomic datasets.

Tool	Does NOT rely on a reference genome database	Uses read coverage	Uses paired-end read mapping	Uses assembly graph	Recovers short repeat regions	Does NOT need an initial binning result	Resolves high-quality and continuous genomes	Code availability	Last updated
Phables	✓	✓	✓	✓	✓	✓	✓	GitHub<br>Bioconda<br>PyPI	2023
FVE-novel (Tithi et al. 2023)	X	✓	✓	X	N/A	✓	✓	GitHub	2020
PHAMB (Johansen et al. 2022)	✓	✓	X	X	✓*	X	X†	GitHub<br>Bioconda	2022
Seeker (Auslander et al. 2020)	✓	X	X	X	X	N/A	X	GitHub<br>PyPI	2021
PPR-Meta (Fang et al. 2019)	✓	X	X	X	✓	N/A	X	GitHub	2021
MARVEL (Amgarten et al. 2018)	✓	✓	X	X	X	X	X	GitHub	2022
MetaPhinder (Jurtz et al. 2016)	X	X	X	X	X	N/A	X	GitHub	2021

† Concatenates multiple sequences without being resolved in the proper order to form a single genome.
* May not correctly associate short repeats with the relevant genomes/MAGs.
N/A: not applicable or not mentioned

2. Bacterial single-copy marker genes

Table S2. List of bacterial single-copy marker genes used to filter components.

Name	Accession	Description
PGK	PF00162.14	Phosphoglycerate kinase
Ribosomal_L23	PF00276.15	Ribosomal protein L23
Ribosomal_L5	PF00281.14	Ribosomal protein L5
Ribosomal_L3	PF00297.17	Ribosomal protein L3
Ribosomal_L6	PF00347.18	Ribosomal protein L6
Ribosomal_S17	PF00366.15	Ribosomal protein S17
Ribosomal_S9	Ribosomal_S9	Ribosomal protein S9/S16
Ribosomal_S8	PF00410.14	Ribosomal protein S8
Ribosomal_S11	PF00411.14	Ribosomal protein S11
Ribosomal_S13	PF00416.17	Ribosomal protein S13/S18
Ribosomal_L10	PF00466.15	Ribosomal protein L10
Ribosomal_L4	PF00573.17	Ribosomal protein L4/L1 family
tRNA-synt_1d	PF00750.14	tRNA synthetases class I (R)
GrpE	PF01025.14	GrpE
Methyltransf_5	PF01795.14	MraW methylase family
TIGR00001	TIGR00001	rpmI_bact: ribosomal protein L35
TIGR00002	TIGR00002	S16: ribosomal protein S16
TIGR00009	TIGR00009	L28: ribosomal protein L28
TIGR00012	TIGR00012	L29: ribosomal protein L29
TIGR00019	TIGR00019	prfA: peptide chain release factor 1
TIGR00029	TIGR00029	S20: ribosomal protein S20
TIGR00043	TIGR00043	TIGR00043: probable rRNA maturation factor YbeY

TIGR00059	TIGR00059	L17: ribosomal protein L17
TIGR00060	TIGR00060	L18_bact: ribosomal protein L18
TIGR00061	TIGR00061	L21: ribosomal protein L21
TIGR00062	TIGR00062	L27: ribosomal protein L27
TIGR00064	TIGR00064	ftsY: signal recognition particle-docking protein FtsY
TIGR00082	TIGR00082	rbfA: ribosome-binding factor A
TIGR00086	TIGR00086	smpB: SsrA-binding protein
TIGR00092	TIGR00092	TIGR00092: GTP-binding protein YchF
TIGR00115	TIGR00115	tig: trigger factor
TIGR00116	TIGR00116	tsf: translation elongation factor Ts
TIGR00152	TIGR00152	TIGR00152: dephospho-CoA kinase
TIGR00158	TIGR00158	L9: ribosomal protein L9
TIGR00165	TIGR00165	S18: ribosomal protein S18
TIGR00166	TIGR00166	S6: ribosomal protein S6
TIGR00168	TIGR00168	infC: translation initiation factor IF-3
TIGR00234	TIGR00234	tyrS: tyrosine--tRNA ligase
TIGR00337	TIGR00337	PyrG: CTP synthase
TIGR00344	TIGR00344	alaS: alanine--tRNA ligase
TIGR00362	TIGR00362	DnaA: chromosomal replication initiator protein DnaA
TIGR00388	TIGR00388	glyQ: glycine--tRNA ligase, alpha subunit
TIGR00389	TIGR00389	glyS_dimeric: glycine--tRNA ligase
TIGR00392	TIGR00392	ileS: isoleucine--tRNA ligase
TIGR00396	TIGR00396	leuS_bact: leucine--tRNA ligase
TIGR00408	TIGR00408	proS_fam_I: proline--tRNA ligase
TIGR00409	TIGR00409	proS_fam_II: proline--tRNA ligase
TIGR00414	TIGR00414	serS: serine--tRNA ligase
TIGR00418	TIGR00418	thrS: threonine--tRNA ligase

TIGR00420	TIGR00420	trmU: tRNA (5-methylaminomethyl-2-thiouridylate)-methyltransferase
TIGR00422	TIGR00422	valS: valine--tRNA ligase
TIGR00435	TIGR00435	cysS: cysteine--tRNA ligase
TIGR00436	TIGR00436	era: GTP-binding protein Era
TIGR00442	TIGR00442	hisS: histidine--tRNA ligase
TIGR00459	TIGR00459	aspS_bact: aspartate--tRNA ligase
TIGR00460	TIGR00460	fmt: methionyl-tRNA formyltransferase
TIGR00468	TIGR00468	pheS: phenylalanine--tRNA ligase, alpha subunit
TIGR00471	TIGR00471	pheT_arch: phenylalanine--tRNA ligase, beta subunit
TIGR00472	TIGR00472	pheT_bact: phenylalanine--tRNA ligase, beta subunit
TIGR00487	TIGR00487	IF-2: translation initiation factor IF-2
TIGR00496	TIGR00496	frr: ribosome recycling factor
TIGR00575	TIGR00575	dnlj: DNA ligase, NAD-dependent
TIGR00631	TIGR00631	uvrb: excinuclease ABC subunit B
TIGR00663	TIGR00663	dnan: DNA polymerase III, beta subunit
TIGR00755	TIGR00755	ksgA: dimethyladenosine transferase
TIGR00810	TIGR00810	secG: preprotein translocase, SecG subunit
TIGR00855	TIGR00855	L12: ribosomal protein L7/L12
TIGR00922	TIGR00922	nusG: transcription termination/antitermination factor NusG
TIGR00952	TIGR00952	S15_bact: ribosomal protein S15
TIGR00959	TIGR00959	ffh: signal recognition particle protein
TIGR00963	TIGR00963	secA: preprotein translocase, SecA subunit
TIGR00964	TIGR00964	secE_bact: preprotein translocase, SecE subunit
TIGR00967	TIGR00967	3a0501s007: preprotein translocase, SecY subunit
TIGR00981	TIGR00981	rpsL_bact: ribosomal protein S12
TIGR01009	TIGR01009	rpsC_bact: ribosomal protein S3

TIGR01011	TIGR01011	rpsB_bact: ribosomal protein S2
TIGR01017	TIGR01017	rpsD_bact: ribosomal protein S4
TIGR01021	TIGR01021	rpsE_bact: ribosomal protein S5
TIGR01024	TIGR01024	rplS_bact: ribosomal protein L19
TIGR01029	TIGR01029	rpsG_bact: ribosomal protein S7
TIGR01030	TIGR01030	rpmH_bact: ribosomal protein L34
TIGR01031	TIGR01031	rpmF_bact: ribosomal protein L32
TIGR01032	TIGR01032	rplT_bact: ribosomal protein L20
TIGR01044	TIGR01044	rplV_bact: ribosomal protein L22
TIGR01049	TIGR01049	rpsJ_bact: ribosomal protein S10
TIGR01050	TIGR01050	rpsS_bact: ribosomal protein S19
TIGR01059	TIGR01059	gyrB: DNA gyrase, B subunit
TIGR01063	TIGR01063	gyrA: DNA gyrase, A subunit
TIGR01066	TIGR01066	rplM_bact: ribosomal protein L13
TIGR01067	TIGR01067	rplN_bact: ribosomal protein L14
TIGR01071	TIGR01071	rplO_bact: ribosomal protein L15
TIGR01079	TIGR01079	rplX_bact: ribosomal protein L24
TIGR01164	TIGR01164	rplP_bact: ribosomal protein L16
TIGR01169	TIGR01169	rplA_bact: ribosomal protein L1
TIGR01171	TIGR01171	rplB_bact: ribosomal protein L2
TIGR01391	TIGR01391	dnaG: DNA primase
TIGR01393	TIGR01393	lepA: GTP-binding protein LepA
TIGR01632	TIGR01632	L11_bact: ribosomal protein L11
TIGR01953	TIGR01953	NusA: transcription termination factor NusA
TIGR02012	TIGR02012	tigrfam_recA: protein RecA
TIGR02013	TIGR02013	rpoB: DNA-directed RNA polymerase, beta subunit
TIGR02027	TIGR02027	rpoA: DNA-directed RNA polymerase, alpha subunit

TIGR02191	TIGR02191	RNaseIII: ribonuclease III
TIGR02350	TIGR02350	prok_dnaK: chaperone protein DnaK
TIGR02386	TIGR02386	rpoC_TIGR: DNA-directed RNA polymerase, beta' subunit
TIGR02387	TIGR02387	rpoC1_cyan: DNA-directed RNA polymerase, gamma subunit
TIGR02397	TIGR02397	dnaX_nterm: DNA polymerase III, subunit gamma and tau
TIGR02432	TIGR02432	lysidine_TilS_N: tRNA(Ile)-lysidine synthetase
TIGR02729	TIGR02729	Obg_CgtA: Obg family GTPase CgtA
TIGR03263	TIGR03263	guanyl_kin: guanylate kinase
TIGR03594	TIGR03594	GTPase_EngA: ribosome-associated GTPase EngA

3. Analysis of PHROG annotations in all known phage genomes

We annotated all the 25,596 complete phage genomes available from the INfrastructure for a PHAge REference Database (INPHARED) (Cook et al. 2021) (as of the May 2023 update) using Prokaryotic Virus Remote Homologous Groups (PHROGs annotation v4). We then selected the annotations belonging to the PHROG categories: *head and packaging*, *connector*, *tail* and *lysis*. Out of 109,404 PHROGs, a total of 2705 PHROGs were selected (head and packaging: 989, connector: 146, tail: 1256 and lysis: 314). Figure S1 denotes the histogram of counts of PHROGs in the phage genomes. The annotation was done using MMSeqs2 (Steinegger and Söding 2017). Figure S1 denotes the histogram of counts of PHROGs in the phage genomes.

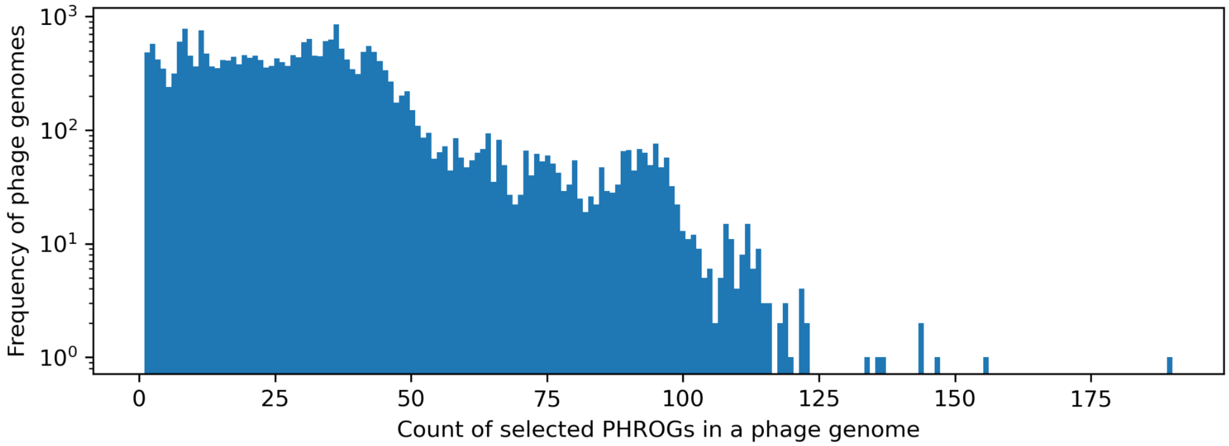

Figure S1. Histograms of counts of PHROGs found in the 25,596 complete phage genomes available from the INfrastructure for a PHAge REference Database.

Using the PHROG hits from the selected categories, we found that,

- 24,533 phage genomes (95.85%) have at least one PHROG hit.
- 24,050 phage genomes (93.96%) have two or more PHROG hits.
- 22,706 phage genomes (88.71%) have five or more PHROG hits.
- 20,319 phage genomes (79.38%) have ten or more PHROG hits.
- 15,152 phage genomes (59.20%) have at least one PHROG from all the categories.
- 24,131 phage genomes (94.28%) have at least one head and packaging PHROG.
- 16,011 phage genomes (62.55%) have at least one connector PHROG.
- 23,162 phage genomes (90.49%) have at least one tail PHROG.
- 21,081 phage genomes (82.36%) have at least one lysis PHROG.

4. Edge confidence

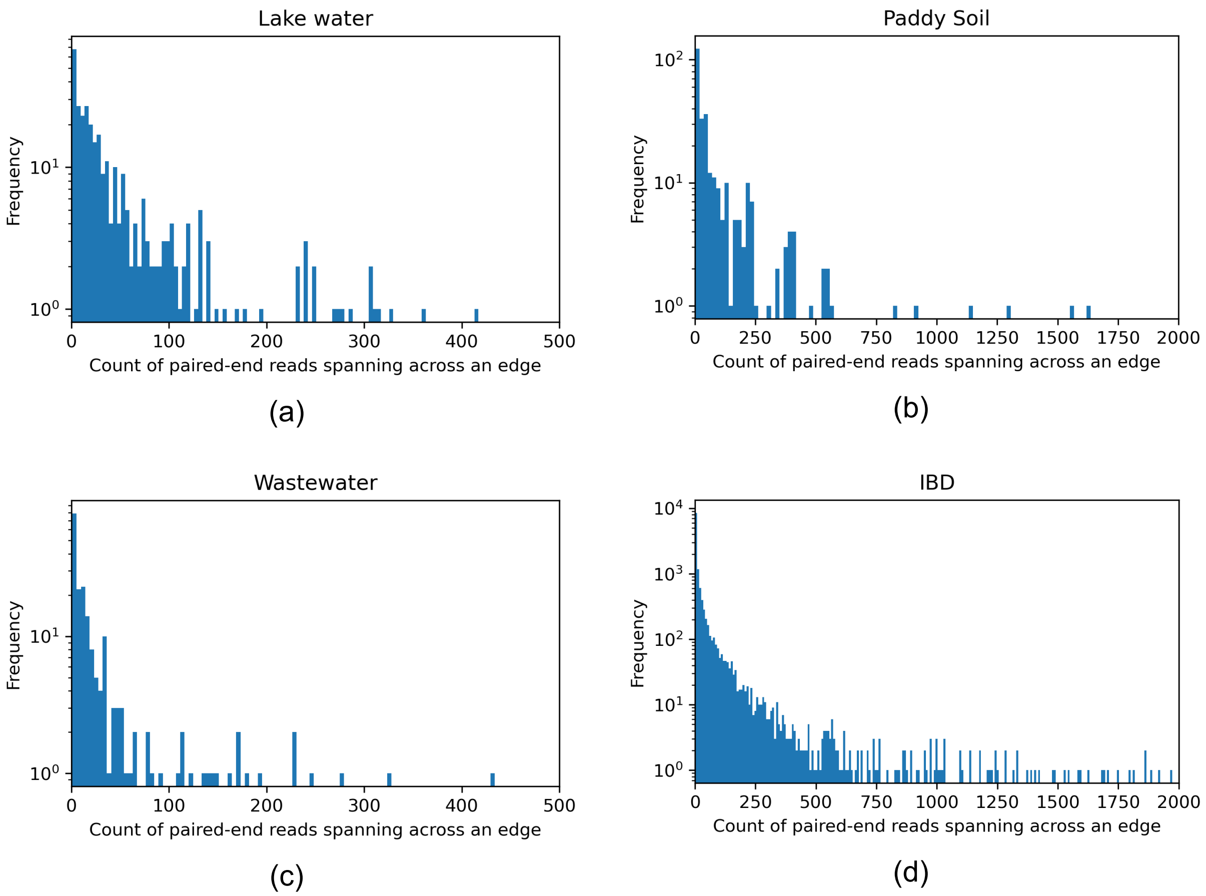

Figure S2. Histograms of paired-end read counts spanning across edges in the assembly graph (edge confidence) for the metagenomic datasets (a) Lake Water, (b) Paddy soil, (c) Wastewater, and (d) IBD.

5. Resolving a case 2 phage component

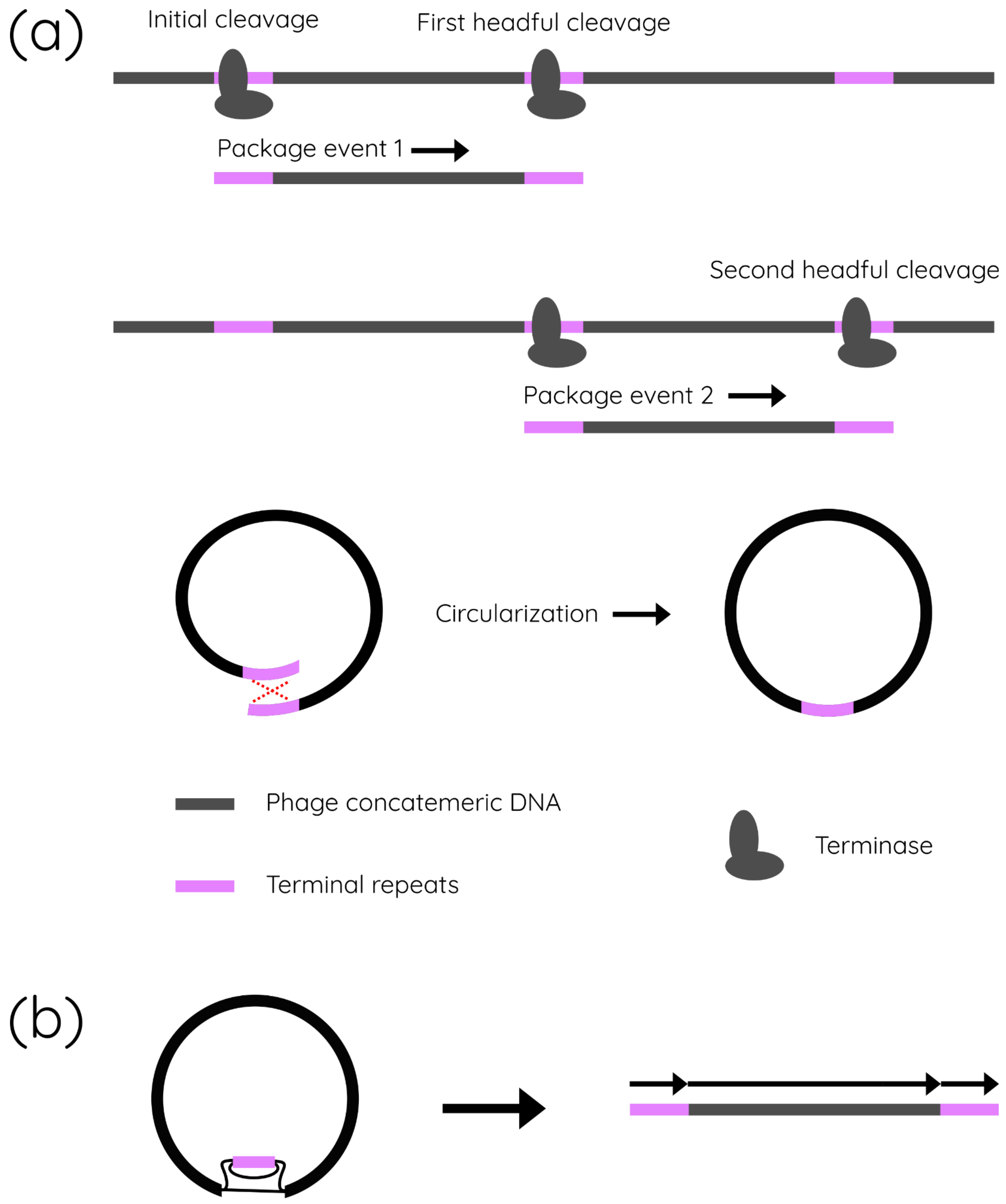

Figure S3: Example of resolving a case 2 phage component. (a) Each packaging event starts and finishes at a packaging recognition site that gets duplicated. The duplicated regions recombine and collapse during circularisation to form a continuous circular genome. (b) Representation of a case 2 phage component with a terminal repeat assembled separately and then resolved into a linearised genome.

6. Algorithms

Algorithm S1: Identify st vertex of G

Input: Assembly graph for a phage bubble (G)
Output: st, the source/sink vertex
Function: $getSourceSink(G)$

```
st_nodes ← ∅
forall source ∈ G.vertices do
    source_is_st ← True
    iteratore ← bfs_layers(G, source)
    last_layer ← list of vertices in the last layer of iterator
    forall v ∈ last_layer do
        v_successors ← G.successor(v)
        if v_successors ≠ ∅ and source ∉ v_successors then
            source_is_st ← False
            break
        if v_successors = ∅ then
            source_is_st ← False
    if source_is_st then
        st_nodes ← st_nodes ∪ source
if st_nodes ≠ ∅ then
    return arg max_{length} st_node
else
    return False
```

7. Information about the datasets

Table S3. Information about the simPhage dataset

Phage genome	Accession number	Simulated coverage (x)
Enterobacteria phage P22	AB426868	100
Enterobacteria phage T7	NC_001604	150
Staphylococcus phage SAP13 TA-2022	ON911718	200
Staphylococcus phage SAP2 TA-2022	ON911715	400

Table S4. Accession numbers of the runs used for each real dataset

Dataset	BioProject	SRA accession numbers
Lake Water	PRJNA756429	SRR15557808, SRR15557809, SRR15557810, SRR15557811, SRR15557812, SRR15557813
Paddy Soil	PRJNA756429	SRR20958178, SRR20958179, SRR20958180, SRR20958181, SRR20958182, SRR2095818
Wastewater	PRJNA434744	SRR6872420, SRR6872421, SRR6872422, SRR6872423, SRR6872424, SRR6872425, SRR6872426, SRR6872427, SRR6872428, SRR6872429, SRR6872430, SRR6872431, SRR6872432, SRR6872433, SRR6872434, SRR6872435, SRR6872436, SRR6872437
IBD (Norman et al. 2015)	PRJEB7772	ERR843912, ERR843913, ERR843914, ERR843915, ERR843916, ERR843917, ERR843918, ERR843919, ERR843920, ERR843921, ERR843922, ERR843923, ERR843924, ERR843925, ERR843926, ERR843927, ERR843928, ERR843929, ERR843930, ERR843931, ERR843932, ERR843933, ERR843934, ERR843935, ERR843936, ERR843937, ERR843938, ERR843939, ERR843940, ERR843941, ERR843942, ERR843943, ERR843944, ERR843945, ERR843946, ERR843947, ERR843948, ERR843949, ERR843950, ERR843951, ERR843952, ERR843953, ERR843954, ERR843955, ERR843956, ERR843957, ERR843958, ERR843959, ERR843960, ERR843961, ERR843962, ERR843963, ERR843964, ERR843965, ERR843966, ERR843967, ERR843968, ERR843969, ERR843970, ERR843971, ERR843972, ERR843973, ERR843974, ERR843975, ERR843976, ERR843977, ERR843978, ERR843979, ERR843980, ERR843981, ERR843982, ERR843983,

		ERR843984, ERR843985, ERR843986, ERR843987, ERR843988, ERR843989, ERR843990, ERR843991, ERR843992, ERR843993, ERR843994, ERR843995, ERR843996, ERR843997, ERR843998, ERR843999, ERR844000, ERR844001, ERR844002, ERR844003, ERR844004, ERR844005, ERR844006, ERR844007, ERR844008, ERR844009, ERR844010, ERR844011, ERR844012, ERR844013, ERR844014, ERR844015, ERR844016, ERR844017, ERR844018, ERR844019, ERR844020, ERR844021, ERR844022, ERR844023, ERR844024, ERR844025, ERR844026, ERR844027, ERR844028, ERR844029, ERR844030, ERR844031, ERR844032, ERR844033, ERR844034, ERR844035, ERR844036, ERR844037, ERR844038, ERR844039, ERR844040, ERR844041, ERR844042, ERR844043, ERR844044, ERR844045, ERR844046, ERR844047, ERR844048, ERR844049, ERR844050, ERR844051, ERR844052, ERR844053, ERR844054, ERR844055, ERR844056, ERR844057, ERR844058, ERR844059, ERR844060, ERR844061, ERR844062, ERR844063, ERR844064, ERR844065, ERR844066, ERR844067, ERR844068, ERR844069, ERR844070, ERR844071, ERR844072, ERR844073, ERR844074, ERR844075, ERR844076, ERR844077, ERR844078, ERR844079, ERR844080, ERR844081

Table S5. Information about the assemblies of all the datasets

Dataset	No. of samples	Read length (bp)	Assembly size (Mb)	Number of contigs	Number of unitigs	Number of edges
simPhage	1	2 × 300	161.19	7	17	19
Lake Water	6	2 × 300	282.52	129 107	129 212	702
Paddy Soil	6	2 × 150	630.03	248 941	249 193	2226
Wastewater	18	2 × 300	53.62	22 035	22 081	135
IBD	171	2 × 250	106.46	23 732	26 329	35 726
Lake Water - metaSPAdes	6	2 × 300	234.88	344 873	887 214	1 121 225
Lake Water - MEGAHIT	6	2 × 300	633.52	1 200 868	N/A*	850 422

* Assembly graphs of MEGAHIT assemblies contain the contig sequences.

8. Commands used

Hecatomb

```
> hecatomb run --reads reads/ --threads 16
```

metaSPAdes

```
> spades.py --meta -1 reads_R1.fastq -2 reads_R2.fastq -o
metaspades_output -t 16 -m 500
```

MEGAHIT

```
# Run MEGAHIT
> megahit -1 reads_R1.fastq -2 reads_R2.fastq -o MEGAHIT -t 16

# Generate FASTG file
> megahit_toolkit contig2fastg 141 final.contigs.fa >
final.contigs.fastg

# Convert FASTG file to GFA
# Using https://github.com/lh3/gfa1/blob/master/misc/fastg2gfa.c
> fastg2gfa final.contigs.fastg > final.contigs.gfa
```

VAMB

```
# Get BAM files
> minimap2 -t 16 -N 50 -ax sr assembly.fasta
reads_sample_n_R1.fastq.gz reads_sample_n_R2.fastq.gz | samtools
view -F 3584 -b --threads 16 > bam_files/reads_sample_n.bam

# Run VAMB
> vamb --cuda --outdir vamb --fasta assembly.fasta --bamfiles
bam_files/*.bam --minfasta 2000
```

PHAMB

```
# Run DeepVirFinder and move results
> python3 ~/DeepVirFinder/dvf.py -i assembly.fasta -o DVF -l
2000 -c 1
> mv DVF/contigs.fna_gt2000bp_dvfpred.txt
annotations/all.DVF.predictions.txt
```

```
# Run Prodigal
> prodigal -i assembly.fasta -d genes.fna -a proteins.faa -p
meta -g 11

# Run hmmsearch
> hmmsearch --cpu {threads} -E 1.0e-05 -o output.txt --tblout
annotations/all.hmmMiComplete105.tbl <micompleteDB> proteins.faa
> hmmsearch --cpu {threads} -E 1.0e-05 -o output.txt --tblout
annotations/all.hmmVOG.tbl <VOGDB> proteins.faa

# Run PHAMB
> run_RF.py assembly.fasta vamb/clusters.tsv annotations
PHAMB_resultdir
```

Phables

```
> phables run --input assembly_graph.gfa --reads fastq_reads
--threads 16
```

CheckV

```
> checkv end_to_end all_sequences.fasta checkv_output -t 16
```

metaQUAST

```
> metaquast.py -R refs/ -o metaquast_output --labels phables
resolved_paths.fasta --threads 16
```

9. Further details about resolved components

Table S6. Length of sequences in case 2 components resolved by Phables

Dataset	Component	Length of longer sequence (bp)	Length of shorter sequence (bp)
Lake Water	1	41 101	525
	2	34 378	618
	3	31 972	672
	4	31 043	522
	5	60 221	504
	6	37 094	944
	7	32 944	505
	8	33 114	508
	Mean	37 734	600
Paddy Soil	1	3327	713
	2	50 917	585
	Mean	27 122	649
Wastewater	1	7930	511
	Mean	7930	511
IBD	1	4310	566
	2	3566	709
	Mean	3938	638

Table S7. The number of components found and resolved for different cases of phage components

Dataset	Case 1 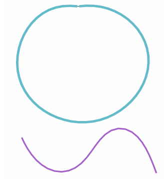	Case 2 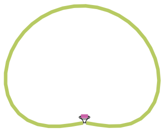	Cae 3 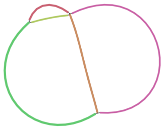	
			Resolved	Found
simPhage	2	0	1	1
Lake Water	192	8	6	6
Paddy Soil	466	2	7	7
Wastewater	92	1	1	1
IBD	262	2	20	32

10. Further benchmarking results

10.1 Bin/genome counts of different quality

Table S8. Benchmarking results of PHAMB (Johansen et al. 2022) and Phables. Best counts are underlined.

Dataset	Tool	Number of phage bins/ genomes identified	Number of genomes with < 10% contamination	Number of genomes with > 90% completeness	Number of complete and high-quality genomes	Number of genomes with CheckV warnings
Lake Water	PHAMB	203	203	162	162	23
	Phables	**210**	**209**	**165**	**165**	**1**
Paddy Soil	PHAMB	189	189	154	154	96
	Phables	**479**	**479**	**389**	**389**	**3**
Wastewater	PHAMB	62	61	40	40	13
	Phables	**94**	**94**	**55**	**55**	**0**
IBD	PHAMB	246	239	193	193	75
	Phables	**307**	**305**	**205**	**205**	**5**
Lake Water - metaSPAdes	PHAMB	168	165	82	82	17
	Phables	**230**	**224**	**182**	**182**	**0**
Lake Water - MEGAHIT	PHAMB	**184**	**183**	98	98	17
	Phables	**184**	182	**156**	**156**	**0**

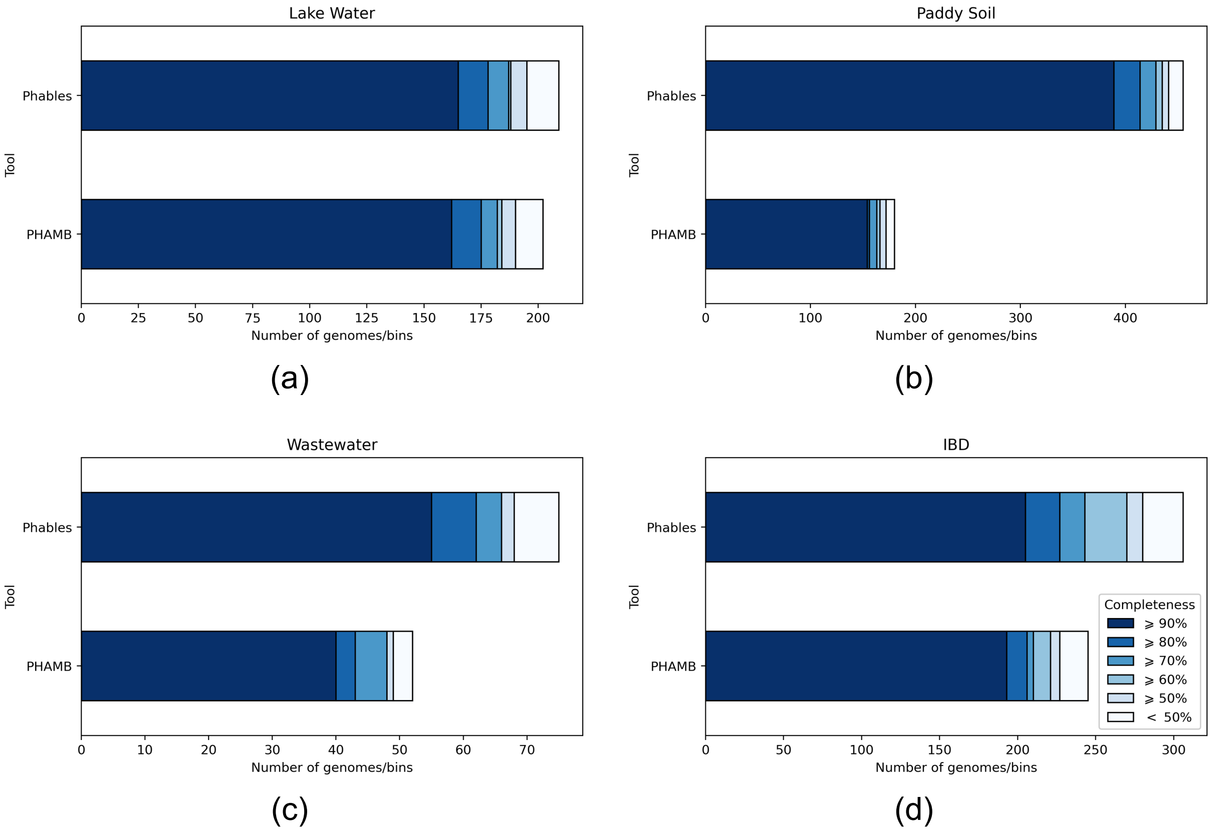

Figure S4: Comparison of CheckV completeness of sequences from Phables and PHAMB (Johansen et al. 2022) for the viral metagenomic datasets (a) Lake Water, (b) Paddy soil, (c) Wastewater, and (d) IBD.

10.2 Quality comparison using all bins containing the selected PHROGs

We benchmarked Phables based on all the PHAMB bins containing the selected PHROGs and the results are presented in Figures S5 - S7. It is clear that even though PHAMB recovers more phage genomes overall, many genomes have CheckV warnings as a result of the erroneous genome structures, high levels of contamination and duplications within genomes due to the presence of multiple closely-related phages. Moreover, it is clear that the majority of the genomes recovered from PHAMB have less than 90% completeness (Figure S7).

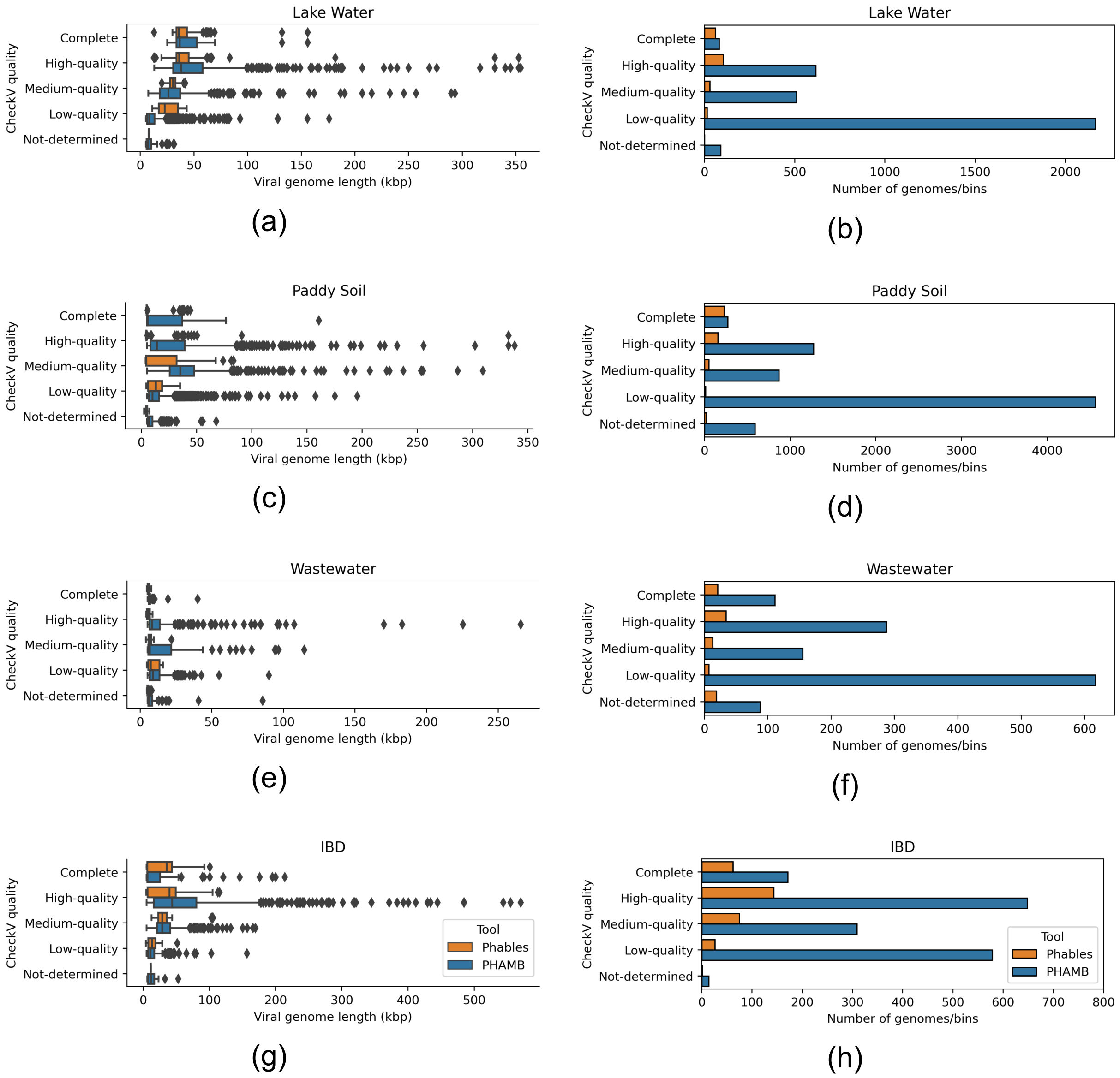

Figure S5: Genome length distribution (first column of figures) and abundance of genomes (second column of figures) belonging to different CheckV quality categories identified by Phables (denoted in orange) and PHAMB (Johansen et al. 2022) (denoted in blue) for the viral metagenomic datasets Lake Water, Paddy soil, Wastewater, and IBD.

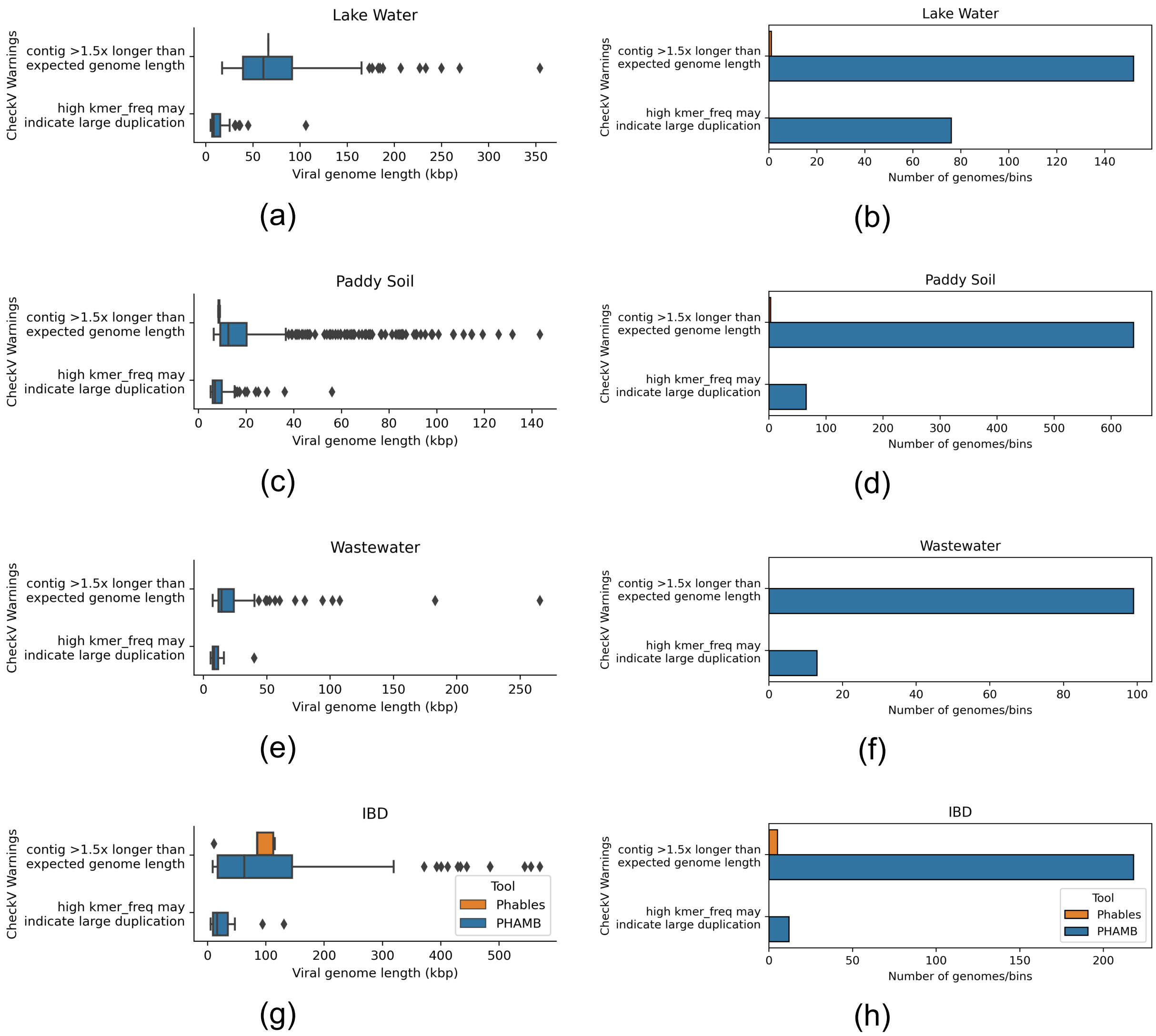

Figure S6: Genome length distribution (first column of figures) and abundance of genomes (second column of figures) having the selected CheckV warnings from Phables (denoted in orange) and PHAMB (Johansen et al. 2022) (denoted in blue) results of the viral metagenomic datasets (a) - (b) Lake Water, (c) - (d) Paddy soil, (e) - (f) Wastewater, and (g) - (h) IBD.

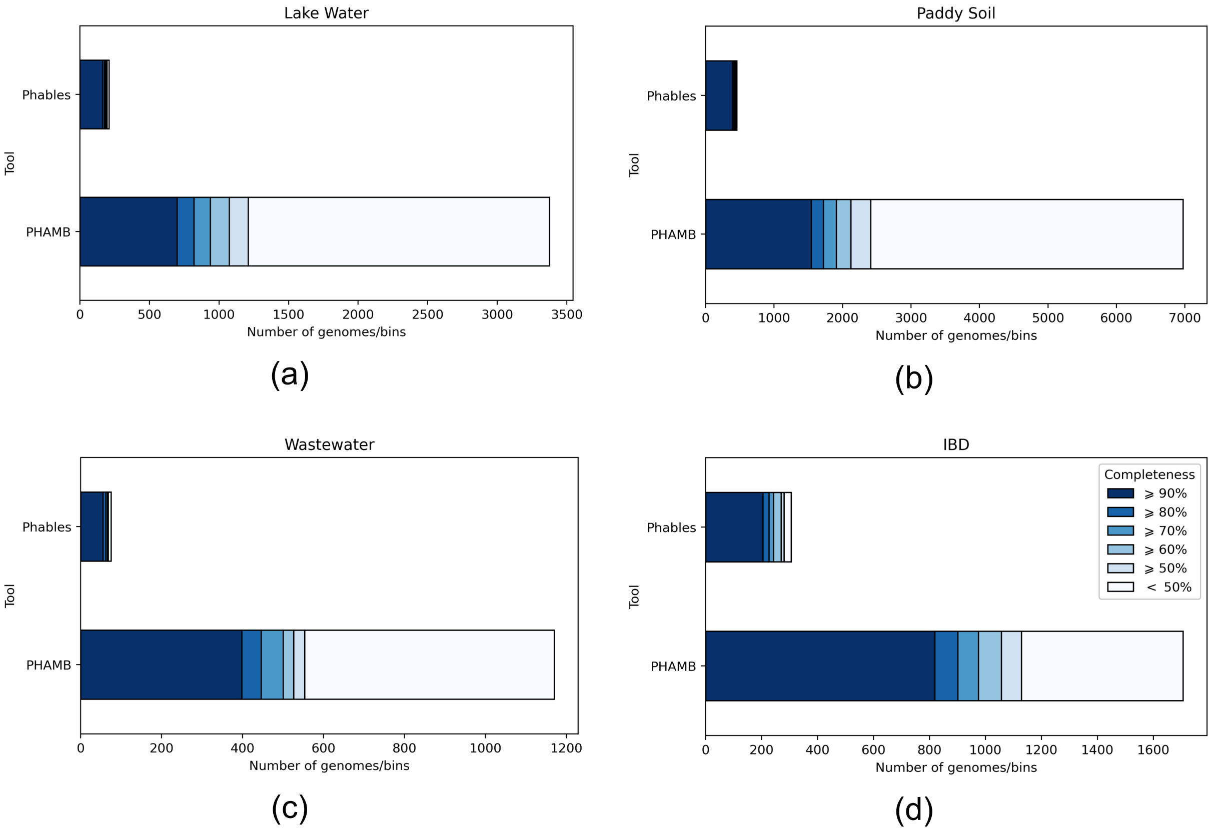

Figure S7: Comparison of CheckV completeness of sequences from Phables and PHAMB (Johansen et al. 2022) for the viral metagenomic datasets (a) Lake Water, (b) Paddy soil, (c) Wastewater, and (d) IBD.

11. Complex case 3 phage components

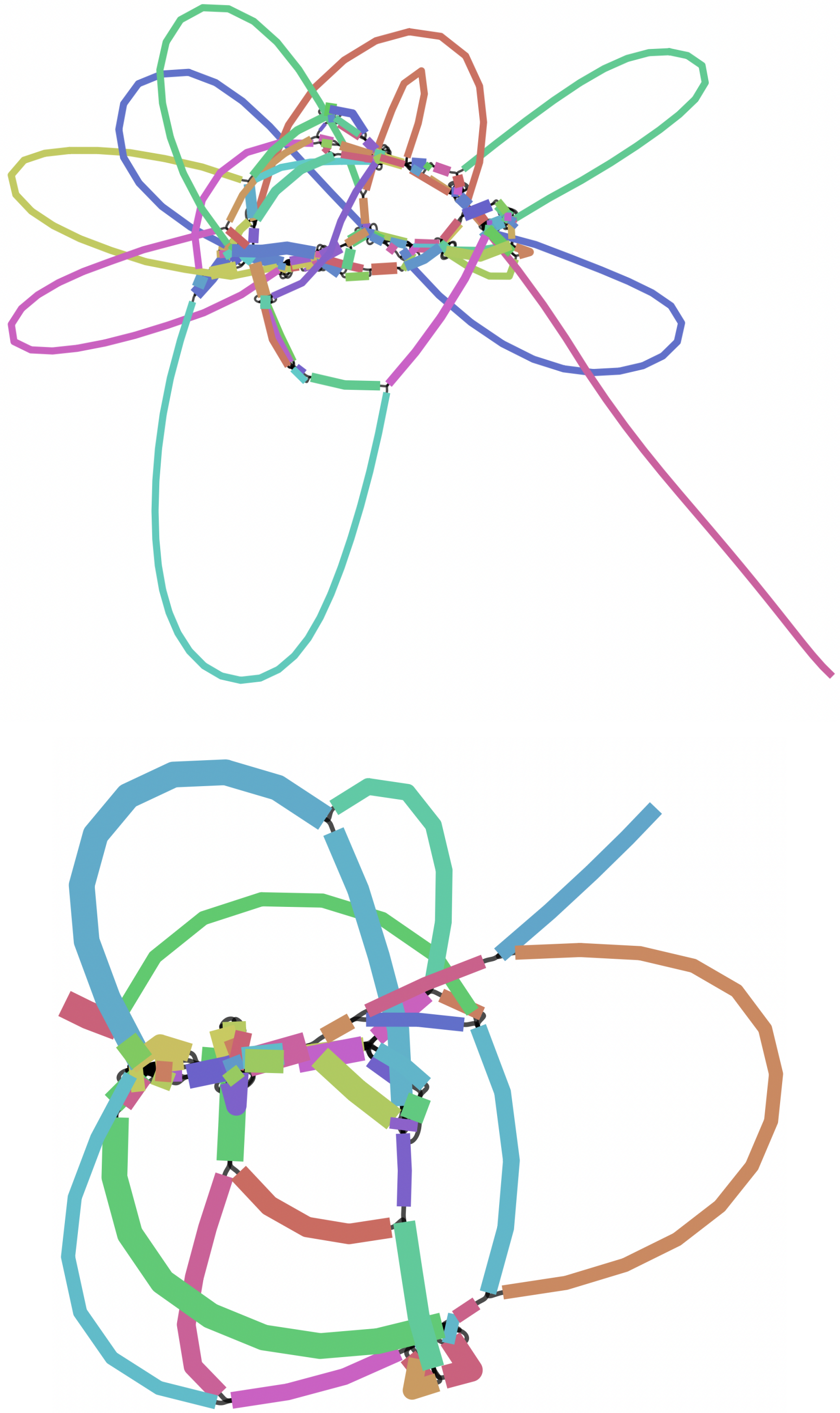

Figure S8. Bandage (Wick et al. 2015) visualisation of two complex case 3 phage components from the IBD dataset where a st vertex could not be found.

12. Similarity analysis of resolved genomes from the IBD dataset

We compared the average nucleotide identity (ANI) of 37 resolved genomes from 14 case 3 phage components of the IBD dataset as it had the most number of case 3 components resolved. All pairs have over 95% ANI, and 20 pairs have over 85% alignment coverage.

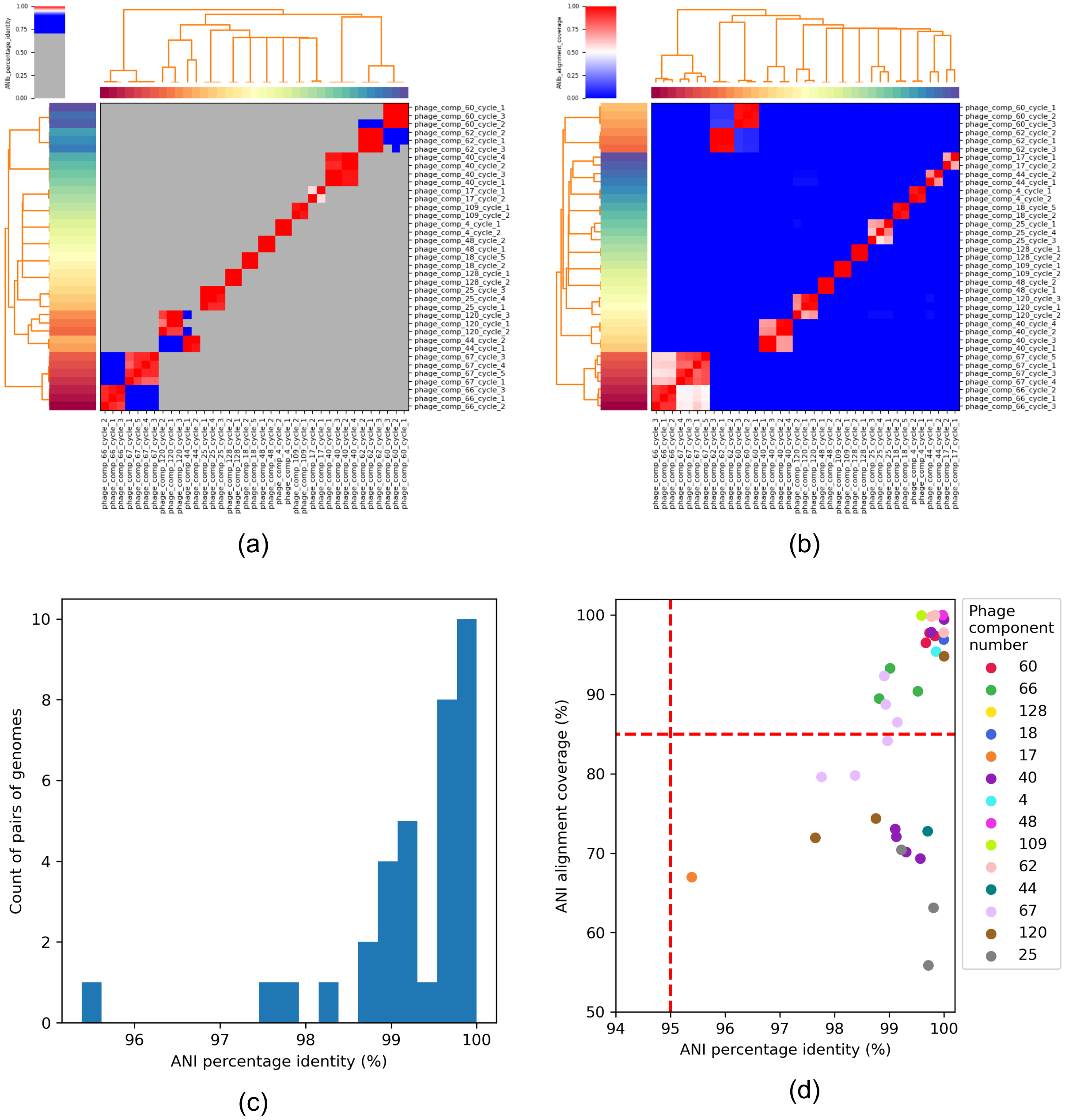

Figure S9. Average nucleotide identity (ANI) analysis of the pairs of resolved genomes from case 3 components of the IBD dataset. (a) ANI identity percentage, (b) ANI alignment coverage, (c) histogram of ANI values and (d) alignment coverage vs. identity percentage.

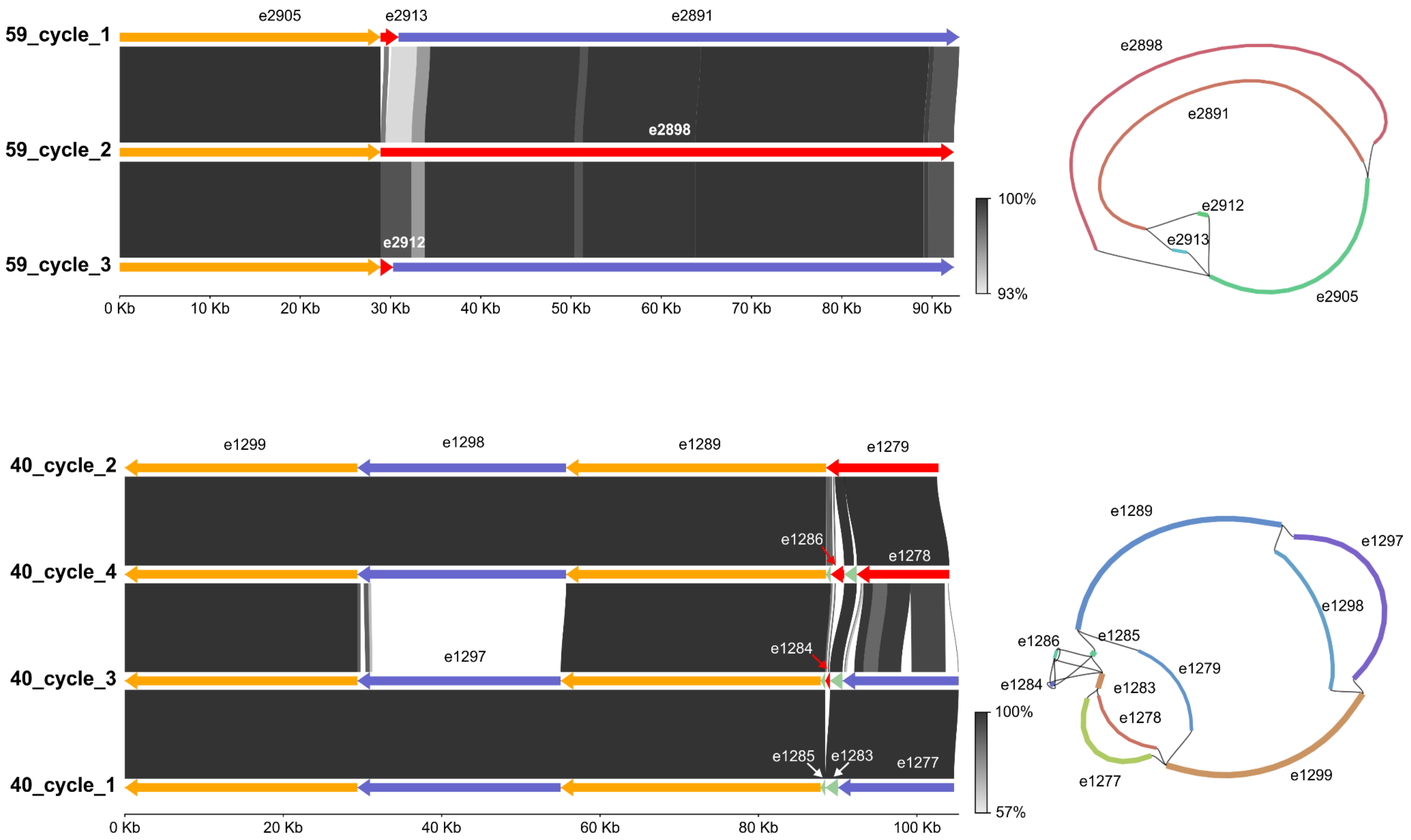

Figure S10: Comparison of similarity between the genomes resolved from two components from the IBD dataset. e[X] denotes the edge numbers from the assembly graph. Red sections denote unique unitigs within the component, blue sections denote unitigs common to two genomes, green sections denote unitigs common to three genomes and orange sections denote unitigs common to all genomes.

13. Results from other assembly methods

We assembled the Lake Water dataset using two popular metagenomic assemblers metaSPAdes (Nurk et al. 2017) and MEGAHIT (Li et al. 2015).

Table S9. The number of components found and resolved for different cases of phage components found in the Lake Water dataset assembled using metaSPAdes and MEGAHIT

Dataset	Case 1 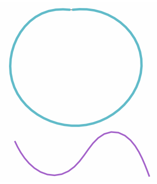	Case 2 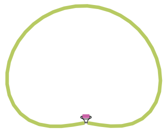	Cae 3 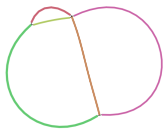	
			Resolved	Found
metaSPAdes	34	0	77	105
MEGAHIT	58	0	75	76

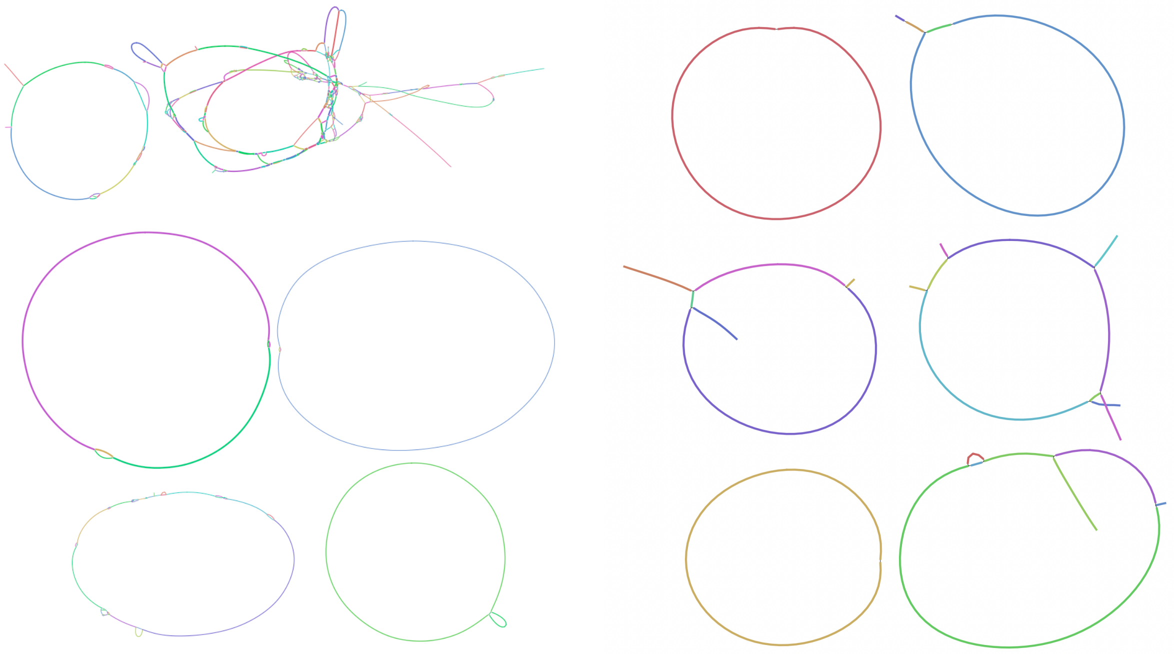

(a) (b)

Figure S11: Bandage visualisations of part of the assembly graph from the Lake Water dataset assembled using (a) metaSPAdes and (b) MEGAHIT.

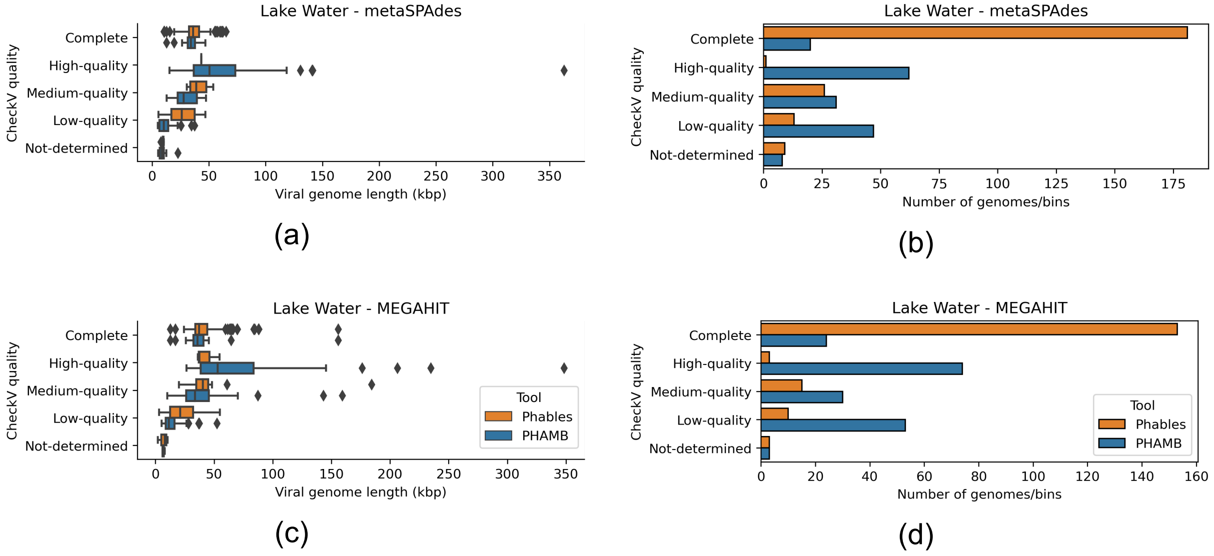

Figure S12: Genome length distribution (first column of figures) and abundance of genomes (second column of figures) belonging to different CheckV quality categories identified by Phables (denoted in orange) and PHAMB (Johansen et al. 2022) (denoted in blue) for the Lake Water dataset assembled using metaSPAdes and MEGAHIT.

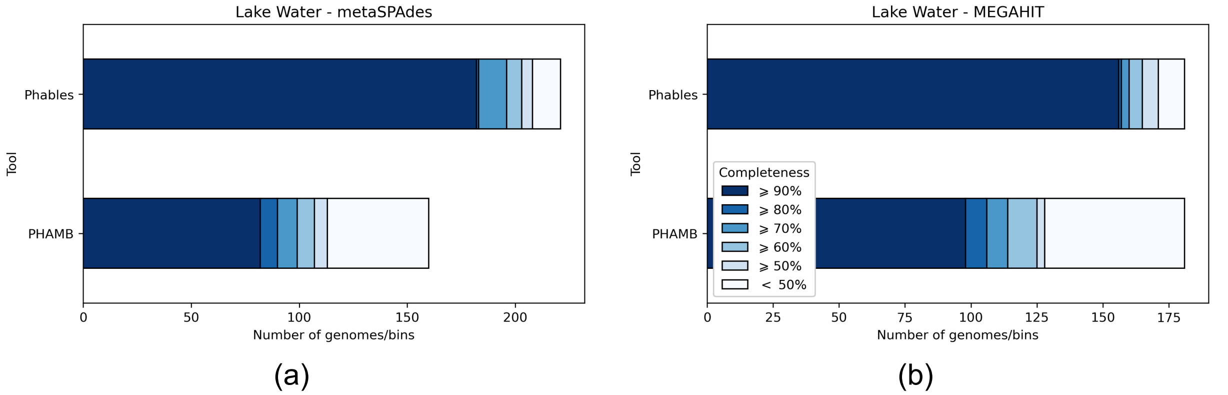

Figure S13: Comparison of CheckV completeness of sequences from Phables and PHAMB (Johansen et al. 2022) results for the Lake Water dataset assembled using (a) metaSPAdes and (b) MEGAHIT.

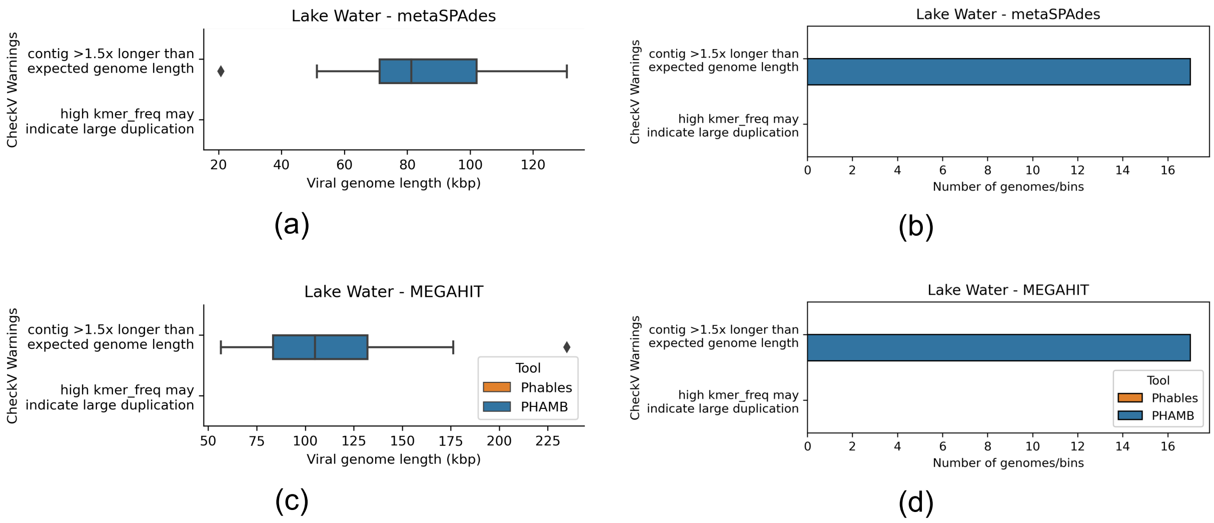

Figure S14: Genome length distribution (first column of figures) and abundance of genomes (second column of figures) having the selected CheckV warnings from Phables (denoted in orange) and PHAMB (Johansen et al. 2022) (denoted in blue) for the Lake Water dataset assembled using metaSPAdes and MEGAHIT.

14. Running time and memory usage

Table S10. Average running time and memory usage of Phables (excluding the preprocessing steps, averaged over 10 runs) with 16 threads for all the datasets. The running time is denoted using minutes (m) and seconds (s), and the memory usage is recorded in megabytes (Mb).

Dataset	Running time (wall time)	Memory usage
Lake Water	9 s	1298.516 Mb
Paddy Soil	12 s	973.806 Mb
Wastewater	5 s	272.720 Mb
IBD	33 s	455.370 Mb
Lake Water - metaSPAdes	1 m 57 s	2325.927 Mb
Lake Water - MEGAHIT	1 m 52 s	3528.125 Mb

Table S11. Average running time and memory usage of the complete Phables workflow (including the preprocessing steps, except assembly, averaged over 10 runs) with 16 threads for all the datasets. The running time is denoted using hours (h), minutes (m) and seconds (s), and the memory usage is recorded in gigabytes (Gb).

Dataset	Running time (wall time)	Memory usage
Lake Water	1 h 8 m 30 s	22.989 Gb
Paddy Soil	3 h 10 m 35 s	43.159 Gb
Wastewater	27 m 18 s	16.309 Gb
IBD	3 h 30 m 51 s	16.403 Gb
Lake Water - metaSPAdes	1 h 58 m 7 s	16.736 Gb
Lake Water - MEGAHIT	2 h 15 m 45 s	52.981 Gb

15. Analysis of PHROG hits in plasmids

We scanned 34,513 plasmids from PLSDB (https://ccb-microbe.cs.uni-saarland.de/plsdb) (Schmartz et al. 2022) against PHROGs and bacterial single-copy marker genes and found that 25,585 plasmids (74.13%) had bacterial single-copy marker genes. We also found that 8928 plasmids (25.87%) had no bacterial single-copy marker genes but had at least one PHROG hit from the categories: head and packaging, connector, tail and lysis (refer to Figure S15 for plasmid counts for the top 40 bacterial genera). The selection of the four PHROG categories ensures that most phages are recovered (as they are conserved in phages), but some plasmids can be misclassified as phage components.

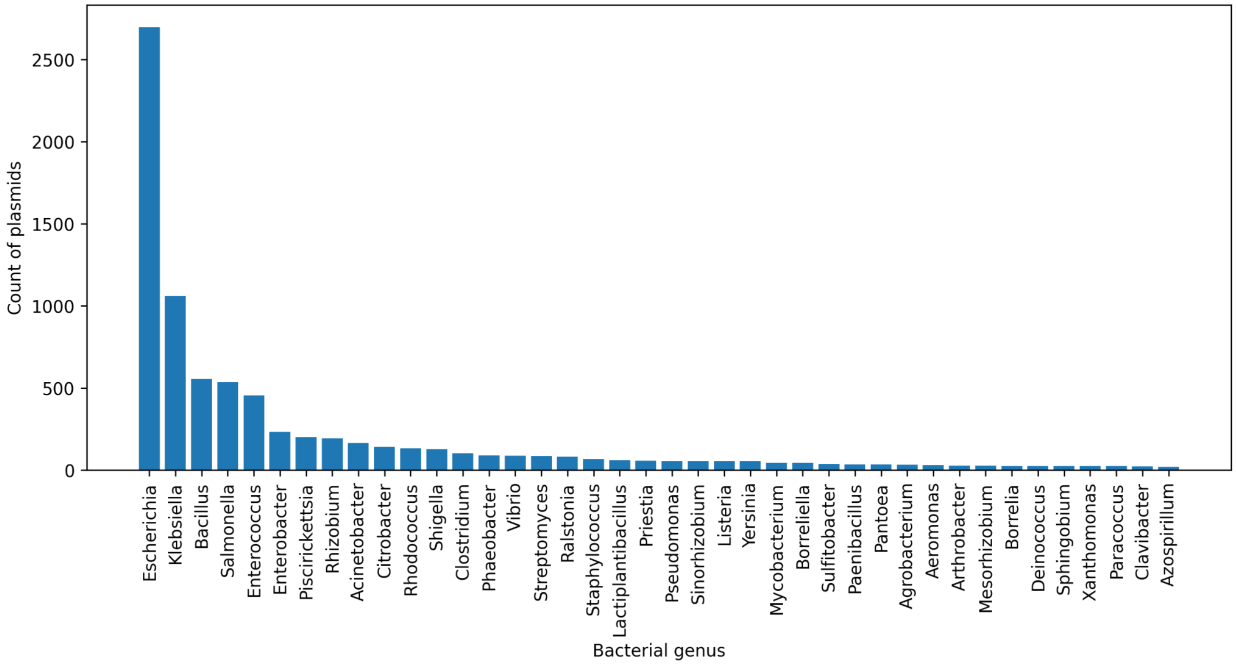

Figure S15: Count of plasmids belonging to the top 40 bacterial genera

Analysis of the selected PHROG categories showed that,

- 705 plasmids (2.04%) have at least one PHROG from all the categories.
- 3793 plasmids (10.99%) have at least one head and packaging PHROG.
- 1052 plasmids (3.05%) have at least one connector PHROG.
- 6988 plasmids (20.25%) have at least one tail PHROG.
- 3393 plasmids (9.83%) have at least one lysis PHROG.

Many temperate phages are found in the host genome as extra-chromosomal plasmids that replicate in line with the cell cycle (Łobocka et al. 2004; Utter et al. 2014; Gilcrease and Casjens 2018), known as *phage-plasmids* (Pfeifer et al. 2021; Pfeifer, Bonnin, and Rocha 2022; Ravin, Svarchevsky, and Dehò 1999), and are elements that are known to be both plasmids and phages (e.g. Escherichia coli bacteriophage P1, Escherichia coli phage N15 or Salmonella phage SSU5). 5363 out of the 8928 plasmids (60.07%) having at least one PHROG hit from the selected PHROG categories are plasmids belonging to bacteria of the genera *Escherichia*, *Enterobacter*, *Salmonella*, *Klebsiella*, *Yersinia*, *Mycobacterium*, *Vibrio*, *Bacillus* and *Clostridiales* (refer to the Figure S16 for plasmid counts of each bacterial genus), which have phages that relate to plasmids encoding proteins homologous to phage sequences (Pfeifer et al. 2021; Gilcrease and Casjens 2018; Dedrick et al. 2016; Octavia, Sara, and Lan 2015; Lan et al. 2009; Myers et al. 2006; Verheust, Fornelos, and Mahillon 2005). Phables can identify such plasmids (or phage-plasmids) as phages and hence, further downstream analysis is required to ensure that the predicted genomes do not include plasmids.

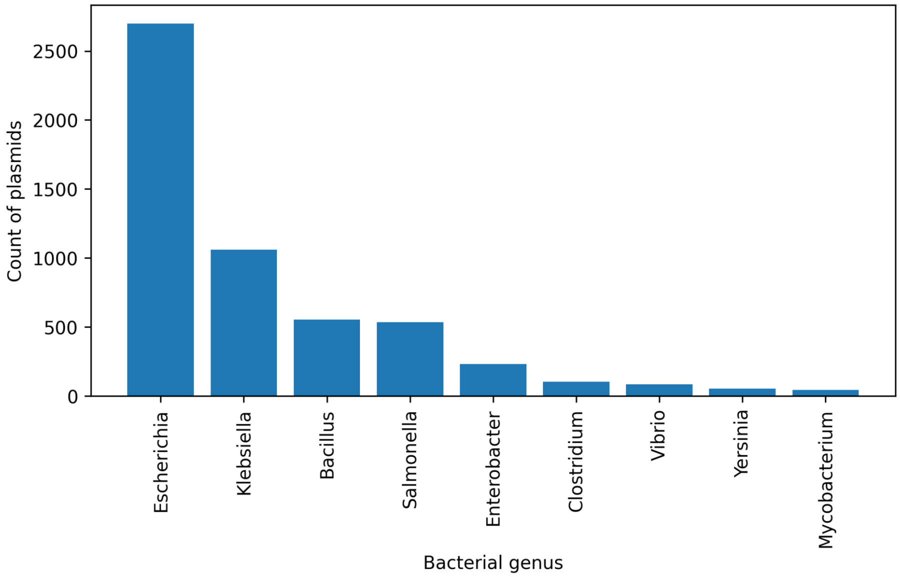

Figure S16: Count of plasmids belonging to bacteria of the genera Escherichia, Salmonella, Klebsiella, Yersinia, Mycobacterium, Vibrio, Bacillus and Clostridiales

References

Amgarten, Deyvid, Lucas P. P. Braga, Aline M. da Silva, and João C. Setubal. 2018. “MARVEL, a Tool for Prediction of Bacteriophage Sequences in Metagenomic Bins.” *Frontiers in Genetics* 9 (August). https://doi.org/10.3389/fgene.2018.00304.

Auslander, Noam, Ayal B. Gussow, Sean Benler, Yuri I. Wolf, and Eugene V. Koonin. 2020. “Seeker: Alignment-Free Identification of Bacteriophage Genomes by Deep Learning.” *Nucleic Acids Research* 48 (21): e121.

Cook, Ryan, Nathan Brown, Tamsin Redgwell, Branko Rihtman, Megan Barnes, Martha Clokie, Dov J. Stekel, Jon Hobman, Michael A. Jones, and Andrew Millard. 2021. “INfrastructure for a PHAge REference Database: Identification of Large-Scale Biases in the Current Collection of Cultured Phage Genomes.” *PHAGE (New Rochelle, N.Y.)* 2 (4): 214–23.

Dedrick, Rebekah M., Travis N. Mavrich, Wei L. Ng, Juan C. Cervantes Reyes, Matthew R. Olm, Rachael E. Rush, Deborah Jacobs-Sera, Daniel A. Russell, and Graham F. Hatfull. 2016. “Function, Expression, Specificity, Diversity and Incompatibility of Actinobacteriophage parABS Systems.” *Molecular Microbiology* 101 (4): 625–44.

Fang, Zhencheng, Jie Tan, Shufang Wu, Mo Li, Congmin Xu, Zhongjie Xie, and Huaiqiu Zhu. 2019. “PPR-Meta: A Tool for Identifying Phages and Plasmids from Metagenomic Fragments Using Deep Learning.” *GigaScience* 8 (6). https://doi.org/10.1093/gigascience/giz066.

Gilcrease, Eddie B., and Sherwood R. Casjens. 2018. “The Genome Sequence of Escherichia Coli Tailed Phage D6 and the Diversity of Enterobacteriales Circular Plasmid Prophages.” *Virology* 515 (February): 203–14.

Johansen, Joachim, Damian R. Plichta, Jakob Nybo Nissen, Marie Louise Jespersen, Shiraz A. Shah, Ling Deng, Jakob Stokholm, et al. 2022. “Genome Binning of Viral Entities from Bulk Metagenomics Data.” *Nature Communications* 13 (1): 1–12.

Jurtz, Vanessa Isabell, Julia Villarroel, Ole Lund, Mette Voldby Larsen, and Morten Nielsen. 2016. “MetaPhinder-Identifying Bacteriophage Sequences in Metagenomic Data Sets.” *PloS One* 11 (9): e0163111.

Lan, Shih-Feng, Chung-Ho Huang, Chuan-Hsiung Chang, Wei-Chao Liao, I-Hsuan Lin, Wan-Neng Jian, Yueh-Gin Wu, Shau-Yan Chen, and Hin-Chung Wong. 2009. “Characterization of a New Plasmid-Like Prophage in a Pandemic Vibrio Parahaemolyticus O3:K6 Strain.” *Applied and Environmental Microbiology*, May. https://doi.org/10.1128/AEM.02483-08.

Li, Dinghua, Chi-Man Liu, Ruibang Luo, Kunihiko Sadakane, and Tak-Wah Lam. 2015. “MEGAHIT: An Ultra-Fast Single-Node Solution for Large and Complex Metagenomics Assembly via Succinct de Bruijn Graph.” *Bioinformatics*. https://doi.org/10.1093/bioinformatics/btv033.

Łobocka, Małgorzata B., Debra J. Rose, Marek Rusin, Arkadiusz Samojedny, Hansjörg Lehnherr, Michael B. Yarmolinsky, and Frederick R. Blattner. 2004. “Genome of Bacteriophage P1.” *Journal of Bacteriology*, November. https://doi.org/10.1128/jb.186.21.7032-7068.2004.

Myers, Garry S. A., David A. Rasko, Jackie K. Cheung, Jacques Ravel, Rekha Seshadri, Robert T. DeBoy, Qinghu Ren, et al. 2006. “Skewed Genomic Variability in Strains of the Toxigenic Bacterial Pathogen, Clostridium Perfringens.” *Genome Research* 16 (8): 1031–40.

Norman, Jason M., Scott A. Handley, Megan T. Baldridge, Lindsay Droit, Catherine Y. Liu, Brian C. Keller, Amal Kambal, et al. 2015. “Disease-Specific Alterations in the Enteric Virome in Inflammatory Bowel Disease.” *Cell* 160 (3): 447–60.

Nurk, Sergey, Dmitry Meleshko, Anton Korobeynikov, and Pavel A. Pevzner. 2017. “metaSPAdes: A New Versatile Metagenomic Assembler.” *Genome Research* 27 (5):

824–34.
Octavia, Sophie, Josephine Sara, and Ruiting Lan. 2015. “Characterization of a Large Novel Phage-like Plasmid in Salmonella Enterica Serovar Typhimurium.” *FEMS Microbiology Letters* 362 (8): fnv044.
Pfeifer, Eugen, Rémy A. Bonnin, and Eduardo P. C. Rocha. 2022. “Phage-Plasmids Spread Antibiotic Resistance Genes through Infection and Lysogenic Conversion.” *mBio* 13 (5): e0185122.
Pfeifer, Eugen, Jorge A. Moura de Sousa, Marie Touchon, and Eduardo P. C. Rocha. 2021. “Bacteria Have Numerous Distinctive Groups of Phage-Plasmids with Conserved Phage and Variable Plasmid Gene Repertoires.” *Nucleic Acids Research* 49 (5): 2655–73.
Ravin, N. V., A. N. Svarchevsky, and G. Dehò. 1999. “The Anti-Immunity System of Phage-Plasmid N15: Identification of the Antirepressor Gene and Its Control by a Small Processed RNA.” *Molecular Microbiology* 34 (5): 980–94.
Schmartz, Georges P., Anna Hartung, Pascal Hirsch, Fabian Kern, Tobias Fehlmann, Rolf Müller, and Andreas Keller. 2022. “PLSDB: Advancing a Comprehensive Database of Bacterial Plasmids.” *Nucleic Acids Research* 50 (D1): D273–78.
Steinegger, Martin, and Johannes Söding. 2017. “MMseqs2 Enables Sensitive Protein Sequence Searching for the Analysis of Massive Data Sets.” *Nature Biotechnology* 35 (11): 1026–28.
Tithi, Saima Sultana, Frank O. Aylward, Roderick V. Jensen, and Liqing Zhang. 2023. “FastViromeExplorer-Novel: Recovering Draft Genomes of Novel Viruses and Phages in Metagenomic Data.” *Journal of Computational Biology: A Journal of Computational Molecular Cell Biology*, January. https://doi.org/10.1089/cmb.2022.0397.
Utter, Bryan, Douglas R. Deutsch, Raymond Schuch, Benjamin Y. Winer, Kathleen Verratti, Kim Bishop-Lilly, Shanmuga Sozhamannan, and Vincent A. Fischetti. 2014. “Beyond the Chromosome: The Prevalence of Unique Extra-Chromosomal Bacteriophages with Integrated Virulence Genes in Pathogenic Staphylococcus Aureus.” *PloS One* 9 (6): e100502.
Verheust, Céline, Nadine Fornelos, and Jacques Mahillon. 2005. “GIL16, a New Gram-Positive Tectiviral Phage Related to the Bacillus Thuringiensis GIL01 and the Bacillus Cereus pBClin15 Elements.” *Journal of Bacteriology*, March. https://doi.org/10.1128/jb.187.6.1966-1973.2005.
